# Molecular subtyping based on immune cell marker genes predicts prognosis and therapeutic response in patients with lung adenocarcinoma

**DOI:** 10.1186/s12885-023-11579-7

**Published:** 2023-11-24

**Authors:** Zi-Tao Liu, Jun-Ting Shen, Yu-Jie Lei, Yun-Chao Huang, Guang-Qiang Zhao, Cheng-Hong Zheng, Xi Wang, Yu-Tian Wang, Long Chen, Zi-Xuan Li, Shou-Zhuo Li, Jun Liao, Ting-Dong Yu

**Affiliations:** 1 Department of Thoracic Surgery, The Third Affiliated Hospital of Kunming Medical University, Kunming, China; 2 Department of Ultrasound, The Third Affiliated Hospital of Kunming Medical University, Yunnan Cancer Hospital, Kunming, China; 3 Department of PET/CT Center, Cancer Center of Yunnan Province, The Third Affiliated Hospital of Kunming Medical University, Yunnan Cancer Hospital, Kunming, China

**Keywords:** Lung adenocarcinoma, Single-cell RNA sequencing, Prognosis, Molecular subtype, Immunotherapy response

## Abstract

**Objective:**

Lung adenocarcinoma (LA) is one of the most common malignancies and is responsible for the greatest number of tumor-related deaths. Our research aimed to explore the molecular subtype signatures of LA to clarify the correlation among the immune microenvironment, clinical outcomes, and therapeutic response.

**Methods:**

The LA immune cell marker genes (LICMGs) identified by single-cell RNA sequencing (scRNA-seq) analysis were used to discriminate the molecular subtypes and homologous immune and metabolic traits of GSE72094 LA cases. In addition, the model-building genes were identified from 1441 LICMGs by Cox-regression analysis, and a LA immune difference score (LIDscore) was developed to quantify individual differences in each patient, thereby predicting prognosis and susceptibility to immunotherapy and chemotherapy of LA patients.

**Results:**

Patients of the GSE72094 cohort were divided into two distinct molecular subtypes based on LICMGs: immune activating subtype (Cluster-C1) and metabolically activating subtype (cluster-C2). The two molecular subtypes have distinct characteristics regarding prognosis, clinicopathology, genomics, immune microenvironment, and response to immunotherapy. Among the LICMGs, *LGR4*, *GOLM1*, *CYP24A1*, *SFTPB*, *COL1A1*, *HLA-DQA1*, *MS4A7*, *PPARG*, and *IL7R* were enrolled to construct a LIDscore model. Low-LIDscore patients had a higher survival rate due to abundant immune cell infiltration, activated immunity, and lower genetic variation, but probably the higher levels of Treg cells in the immune microenvironment lead to immune cell dysfunction and promote tumor immune escape, thus decreasing the responsiveness to immunotherapy compared with that of the high-LIDscore patients. Overall, high-LIDscore patients had a higher responsiveness to immunotherapy and a higher sensitivity to chemotherapy than the low-LIDscore group.

**Conclusions:**

Molecular subtypes based on LICMGs provided a promising strategy for predicting patient prognosis, biological characteristics, and immune microenvironment features. In addition, they helped identify the patients most likely to benefit from immunotherapy and chemotherapy.

**Supplementary Information:**

The online version contains supplementary material available at 10.1186/s12885-023-11579-7.

## Introduction

Lung cancer causes the majority of cancer-associated deaths globally [1] and was responsible for almost one-quarter of all cancer deaths in 2020 [2]. Lung adenocarcinoma (LA) is the most prevalent subtype of lung cancer. The main treatment for early-stage LA is surgical resection. However, a few patients are prone to recurrence [3], and 80–85% of patients lose the opportunity for surgery at the time of diagnosis [4]. Chemotherapy, radiotherapy, and combined treatment have been used for the clinical treatment of LA patients who have lost the occasion for operation. However, the long-term survival rates of most LA patients remain unsatisfactory [5, 6]. In addition, the negative effects of chemotherapeutic medications and the high resistance rate of targeted drugs have affected LA treatment. Fortunately, immunotherapy is becoming one of the most anticipated treatments for LA [7]. However, different patients have completely different responses to the effects of immunotherapy [8], which probably reflect the differences in T-cell function and tumor immunogenicity, and also intra-tumor heterogeneity. Therefore, exploring differences in the tumor immune microenvironment (TIME) is critical to determine which patients respond to immunotherapy.

With bulk RNA sequencing, different cells in the tumor microenvironment (TME) were all sequenced together. Consequently, they cannot distinguish heterogeneity, thus masking the key signals and molecular events specific to different cell subtypes in the TME [9, 10]. However, single-cell RNA sequencing (scRNA-Seq) offers the opportunity to independently analyze individual cells or individual subpopulations to reveal tumor heterogeneity, predict prognosis, and dynamically analyze cellular differentiation processes [11]. Current studies on the TME have demonstrated that the interaction between untransformed immune cells or stromal cells and tumor cells promotes tumor development in the early stage and also plays a crucial role in the late-stage or metastatic stage [12]. The degree of immune cell infiltration and its functional status in cancer tissue significantly impact patients’ prognosis and treatment response [13]. Furthermore, the current study shows that molecular subtypes based on TME identification have significant advantages in predicting patient responsiveness to immunotherapy [14].

In this study, we combined scRNA-seq with bulk RNA-seq of TCGA and GEO to assess molecular subtypes associated with the TIME in LA, clarifying the characterization of immune and metabolic features among different molecular subtypes. Then, survival-associated genes were further sifted through univariate Cox and LASSO regression analysis, and a LIDscore model was constructed to quantify the individual differences of LA patients and accurately forecast the prognosis of patients and the sensitivity of patients to immunotherapy and chemotherapy.

## Materials and methods

### Collection and processing of samples and data

The cancer tissues from two patients with LA were obtained from the Third Affiliated Hospital of Kunming Medical University (Kunming, China), and single-cell isolation and high-throughput sequencing was performed by NoveBio (Shanxi, China). Using cellranger, sequencing quality was controlled, and reads with low sequencing quality were removed. The number of reads and sequencing quality measured by the sample was preliminarily counted. Then, the reads were compared with the reference genome and annotated as specific genes. After UMI was corrected and counted, the unfiltered feature-barcode matrix was obtained. In accordance with the unfiltered feature-barcode matrix, cellranger authenticates and differentiates cells and non-cells in the data. Finally, we obtained scRNA-Seq data of 24,964 cells. The Institutional Research Ethics Committee approved this study, and informed consent from the patients was obtained.

An independent cohort of 442 LA simple sequences GSE72094 and transcriptomic and clinical data were downloaded from the GEO database (https://www.ncbi.nlm.nih.gov/geo/). In addition, transcriptomic RNA-sequencing data, mutational information, and corresponding clinicopathological features of LA patients in TCGA were downloaded from Genome Data Sharing Data Portal (https://portal.gdc.cancer.gov/), which included 594 samples (535 cancer samples and 59 normal samples).

### Processing and dimensionality reduction of scRNA-seq data and extraction of LA immune cell marker genes (LICMGs)

scRNA-seq expression data were initially processed by the “Seurat” R package [15]. The percentage of mitochondrial genes is calculated by the PercentageFeatureSet function, and the relationship between sequencing depth and mitochondrial gene sequences is calculated by correlation analysis. Cells with a mitochondrial gene content < 5% and sequencing numbers > 50 were selected as screening conditions. The scRNA-seq expression data were then standardized using the LogNormalize method, and the top 1,500 genes with large cell-to-cell coefficients of variation were extracted by the FindVariableFeatures method.

For the 1,500 hypervariable genes screened by the appeal, the principal component analysis (PCA) algorithm was used to select the top 15 dimensions with P < 0.05, and then the t-distributed random neighborhood embedding (t-SNE) algorithm was used for dimension reduction. Dimensionality reduction cluster classification analysis was performed to obtain primary clusters. Each cluster was then subjected to differential expression analysis by the “limma” package with a cutoff criterion of log2(FC) ≥ 1 and an adjusted P-value of < 0.05. The top 10 most significantly different marker genes in each cluster were used to create a heatmap. Based on the marker genes in each cluster, identification and annotation of each cluster were done by the “SingleR” R package [16] and validated and corrected using the CellMarker database. After completing the cluster annotation, differential analysis was performed on each cell cluster, and the LA immune cell marker genes (LICMGs) were selected for subsequent analysis.

### Gene ontology (GO) and kyoto encyclopedia of genes and genomes (KEGG) analysis

The GO and KEGG analyses were employed to identify the potential functions and pathways that LICMGs are involved in, while the significant ones were visualized by the “GOplot” R package [17].

### Unsupervised clustering of LA samples based on LICMGs

Unsupervised consensus clustering was performed on the GSE72094 dataset using LICMGs based on the k-means clustering algorithm in the “ConsensusClusterPlus” package in R [18]. K ranges from 2 to 9, and the optimal number of clusters is determined based on the consensus score and cumulative distribution function (CDF). Furthermore, Kaplan–Meier survival analysis (log-rank test) was applied to verify the performance of various clusters to predict survival differences.

### Gene set variation analysis (GSVA)

A gene set of 186 KEGG pathways was obtained from the MSigDB database (https://www.gsea-msigdb.org/gsea/ MSigDB /). The “GSVA” R package [19] was used to calculate the GSVA score of each patient in the GSE72094 dataset and analyze the differences of all pathways among different groups according to the unsupervised clustering results. adj.P.val < 0.05 was used as the filtering condition to find the significantly different pathways. The “PheATmap” package was used to draw the heatmap of the 20 pathways with the most significant difference.

### Assessment of tumor sample purity and immune cell ratio

CIBERSORT [20] was used to perform cell type enrichment analysis of different immune cell types based on somatic RNA-seq data to infer the composition of different immune cells [21]. We estimated relative infiltration fractions of 22 immune cell types in the TME of GSE72094 dataset samples by CIBERSORT. At the same time, we obtained the characteristic gene sets of 28 immune cells from recent publications [22] and quantified the relative infiltration of 28 immune cells in different groups of TME by single-sample gene set enrichment analysis (ssGSEA).

The ESTIMATE algorithm [23] was used to assess the TME components of a sample, including ImmuneScore, StromalScore, and Tumor Purity. Based on the GSE72094 dataset, we used the ESTIMATE method to infer Tumor Purity and the presence of infiltrating immune/stromal cells in tumor tissue.

### Construction of the LIDscore model

LICMGs were continued as a candidate hub gene, and the differentially expressed genes were identified and screened out based on the TCGA dataset, and the prognostic genes were screened out by univariate survival analysis. Univariate Cox values with a P-value < 0.05, and logFC > 1.75 were used as the screening condition to obtain candidate genes associated with survival. As candidates, genes with P < 0.05 were chosen, and the number of predictors was decreased using LASSO regression to eliminate collinearity among these genes. Cross-validation determines the best lambda for the model and outputs the model formula. The formula is as follows:

$$\begin{array}{l}{\rm{LIDscore = }}\left( {{\rm{expICMG1 \times coef1}}} \right){\rm{ + }}\left( {{\rm{expICMG2 \times coef2}}} \right)\\{\rm{ + }}...{\rm{ + }}\left( {{\rm{expICMGn \times coefn}}} \right){\rm{.}}\end{array}$$

LIDscores were validated using the GSE72094 dataset. The PCA was performed to assess the inter-group discrimination and intra-group similarity of LIDscore in the training and validation cohort. The predictive ability of LIDscore in the training and validation cohort was appraised by receiver operating characteristic (ROC) curve analysis. The potential of LIDscore as an independent prognostic factor was assessed using univariate and multivariate Cox-regression analysis.

### Assessment of TIME

XCell [24], TIMER [25], quanTIseq [26], MCP-counter [27], EPIC [28], CIBERSORT-abs, and CIBERSORT [20, 21, 29] were performed to estimate immune cell infiltration levels to assess the proportion of immune cells in each LA samples in TCGA. In addition, the richness of 29 immune features in each LA sample was quantified by the ssGSEA score.

### Assessment of clinicopathological characteristics

The distribution of clinicopathological characteristics in different subgroups was compared and visualized by the “pheatmap” R package. At the same time, the distribution of immune subtypes in different subgroups was compared, which determined and characterized six immune subtypes across multiple cancer types based on an extensive immune genomic analysis of more than 10,000 tumors using TCGA pooled data type.

### Assessment of molecular characteristics

Gene Set Enrichment Analysis (GSEA) evaluated microarray data at the gene set level. We used the gene set (c2.cp.kegg.v7.4.symbols.gmt) as the internal reference gene set, with P < 0.05 as the screening conditions, to screen the important signaling pathways enriched in different subgroups. Correlation analysis was carried out between LIDscore and tumor mutation burden (TMB). Based on the relevant mutation information of LA samples from the TCGA database, the “Maftools”[30] package in R was used to analyze the somatic variants of different subgroups of samples comprehensively.

### Evaluation of the efficacy of immunotherapy and drug sensitivity

To assess the prognostic value of immunotherapy therapy in different subgroups, each LA sample in the TCGA cohort had its tumor immune dysfunction and exclusion (TIDE) scores calculated online (http://tide.dfci.harvard.edu/) to predict a patient’s response to immune checkpoint inhibitors [31]. The ability of LIDscore is assessed by the ROC curve and AUC value and compared with the TIDE scores. Meanwhile, tumor inflammation signature (TIS) [32], which is used to predict anti-PD-1 responses, was also compared with the LIDscore.

To assess the response of different subgroups to chemotherapy, we screened the drug using the “pRRophetic” R package [33] and evaluated the Half inhibitory concentration (IC50) to assess the efficacy of the drug (pFilter = 0.001 and corPvalue = 0.001).

### Independent analysis of model genes

To gain a deeper understanding of the prognostic model, for each model gene, we used cellular localization analysis based on single-cell data, differentially expressed analysis in paired tumor and non-tumor samples, and predictive survival ability based on TCGA data. The immunohistochemical result of the protein of each model gene was obtained from the Human Protein Atlas (HPA) (www.proteinatlas.org). Those genes that could not be found in HPA were analyzed by immunohistochemical (IHC) staining with 20 paired tumor and non-tumor samples obtained from the Third Affiliated Hospital of Kunming Medical University (Kunming, China). Methods are as follows: xylene was used to deparaffinize sections, which were then immersed in EDTA antigen extraction buffer for antigen retrieval, blocked with 3% hydrogen peroxide, incubated with rabbit polyclonal antibody to IL7R (1:100; biorbyt; P16871) overnight, followed by secondary antibodies incubation and staining with DAB. Two experienced pathologists blinded to all raw data assessed the immunohistochemical staining results. The evaluation criteria for IHC staining results are the same as those used in HPA.

### Statistical analysis

Single-cell data analysis was performed in R language 4.1.3, and the rest of the statistical analysis and result presentation were performed in R language 4.2.0 (http://www.r-project.org). Therefore, P-values < 0.05 are deemed to be statistically significant.

## Results

### scRNA-seq analysis to identify the type of LA Cells and select LICMGs

**Fig. 1:**
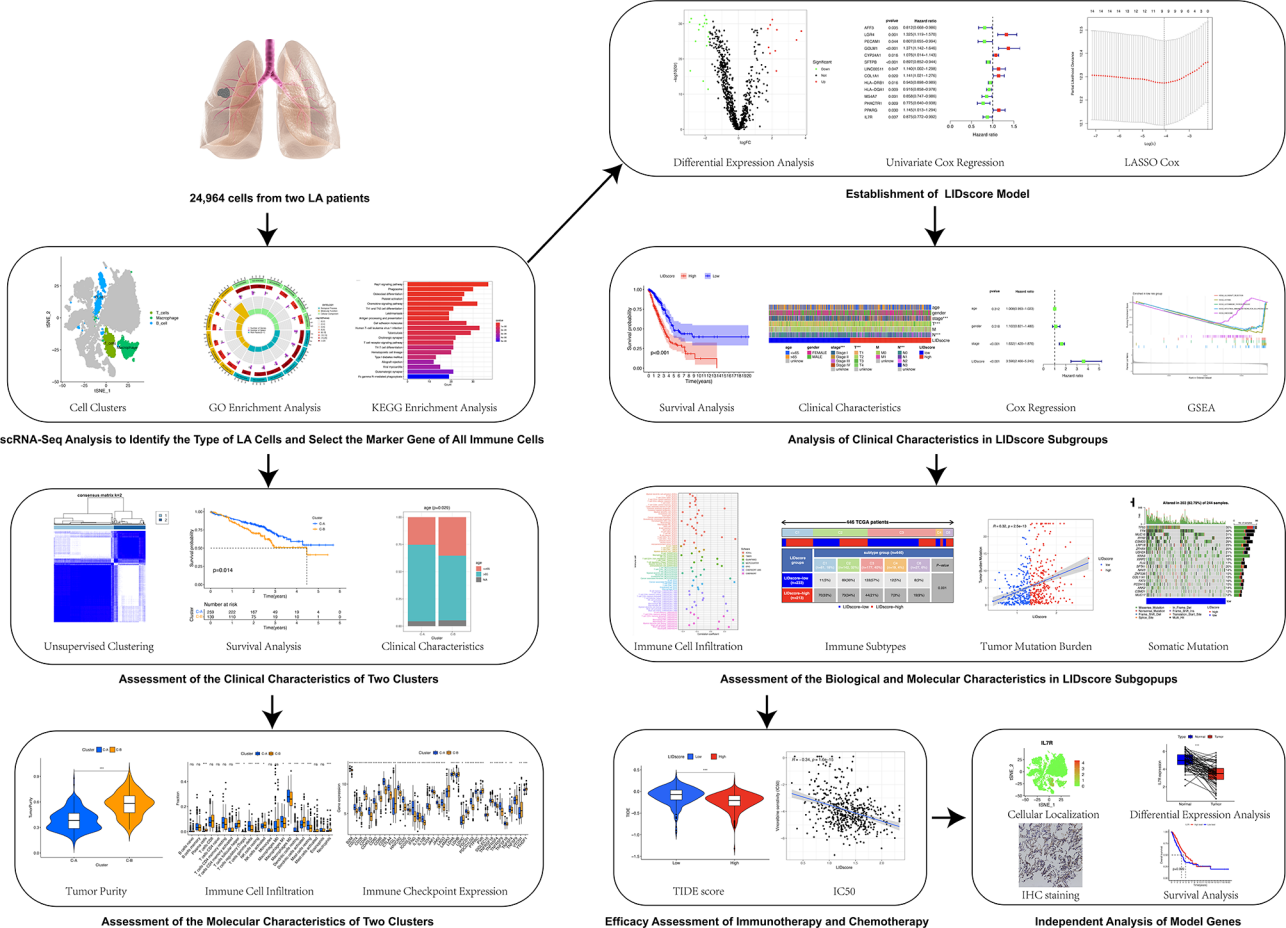
Schematic illustration of the study design

**Fig. 2:**
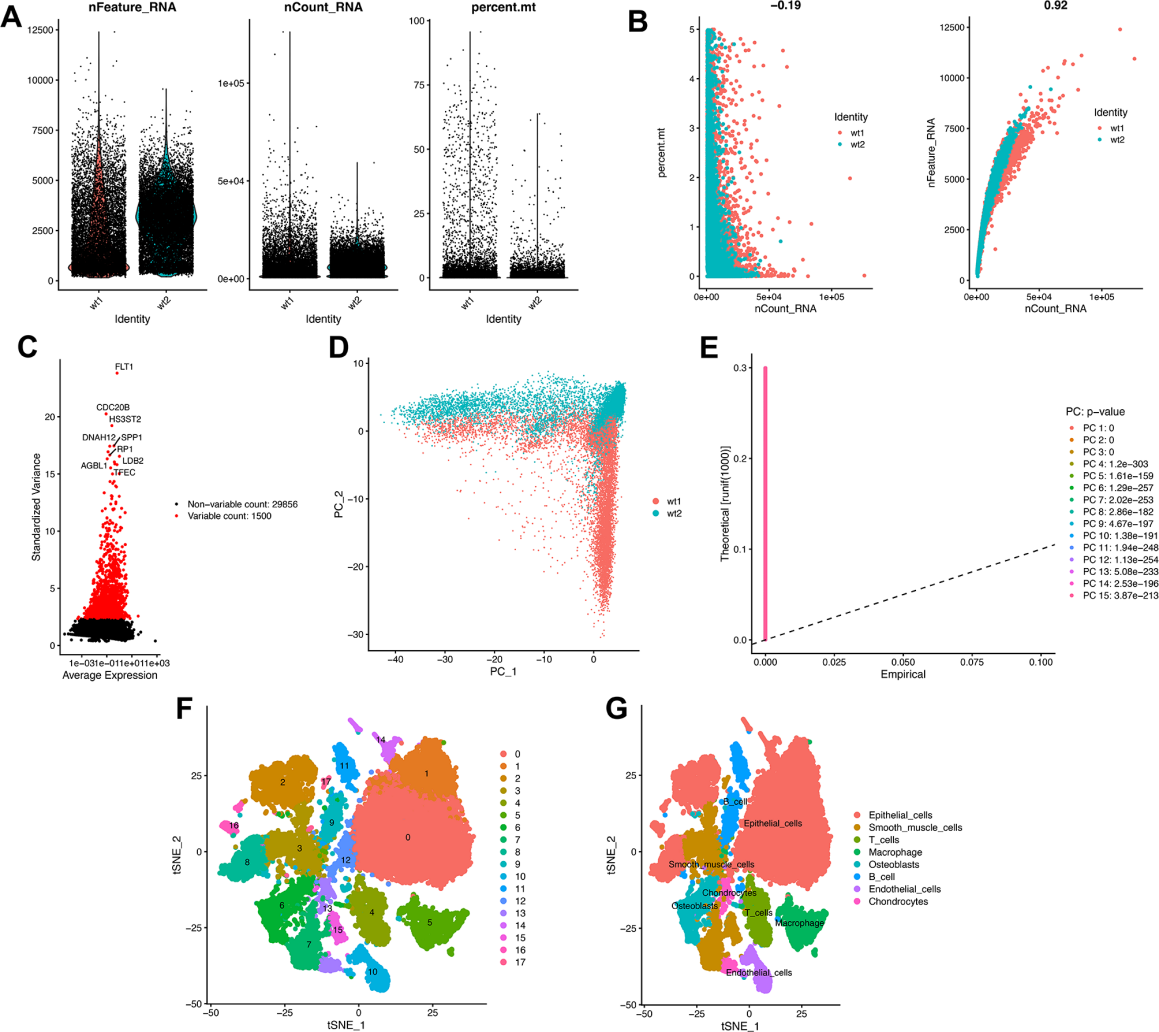
scRNA-seq analysis identified LICMGs in patients with LA. (**A**) The analysis comprised a total of 24,964 cells from two LA patients. Quality control, including the sequencing depth (left), number of identified genes (middle), and percentage of mitochondria genome (right) in each single-cell sample. (**B**) The correlation between sequencing depth and the proportion of mitochondrial genes is shown by a dot plot (left), as is the correlation between sequencing depth and gene expression (right). (**C**) The variance diagram (red dots) for LA cells depicts 1500 highly variable genes. The black dots signify genes without significant variation. Among them, the abscissa is the average expression level of genes, and the ordinate is the standardized variance. (**D**) Principal component analysis (PCA) showed that the LA cells were not separated. (**E**) PCA found the first 15 principal components with a P-value < 0.05. (**F**, **G**) The t-SNE technique categorized the 18 primary components, and eight-cell clusters were annotated

Figure 1 shows the schematic illustration of the study design. Twenty-four thousand nine hundred sixty-four cells from two LA samples were quality-controlled and normalized with a filtration condition for a number of genes fewer than 50 and a number of mitochondria greater than 5% (Fig. 2A). Subsequently, we found that the detection of sequencing depth was negatively correlated with mitochondrial content (R=-0.19) and positively correlated with the number of genes (R = 0.92; Fig. 2B). After quality was controlled and normalized, a total of 31,356 genes were obtained, and the 1,500 genes with the most significant differences were selected as highly variable genes for subsequent analysis (Fig. 2C).

Dimensionality reduction of scRNA-seq data was performed using principal component analysis (PCA), which did not cause significant separation between LA cells (Fig. 2D). We performed PCA on the two single-cell samples and selected the top 15 PCs with the smallest P-value for subsequent analysis (Fig. 2E). The top 20 genes significantly associated with PCs are shown in **Figure **S1**A**, and we show the first four PCs. In order to obtain more accurate clustering of cell samples, according to the t-SNE algorithm, 18 different clusters were further subdivided (Fig. 2F). Differential expression analysis identified a total of 6,627 marker genes in 18 clusters, and the top 10 genes in each cluster were presented as heatmaps (**Figure **S1**B)**. The annotation result of 18 clusters passing through the “SingleR” R package is shown in Fig. 2G. The marker gene of all immune cells was selected for subsequent analysis. Finally, 1,441 LICMGs were identified in LA.

### GO and KEGG analysis of LICMGs

**Fig. 3:**
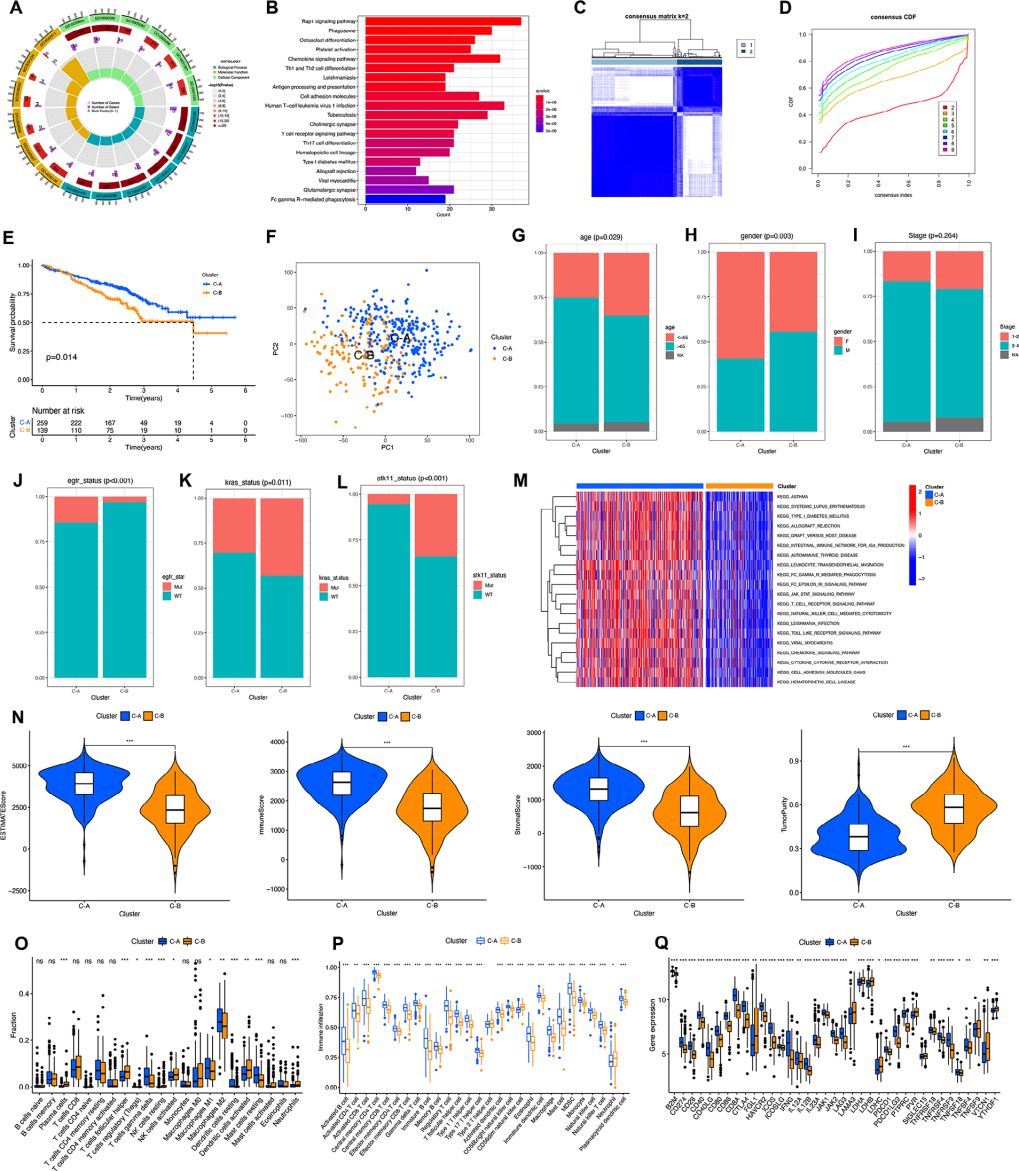
Unsupervised clustering analyses and characteristics of two clusters (**A**, **B**) GO and KEGG enrichment analysis of HDRGs. (**C**) Consensus clustering matrix for k = 2. (**D**) CDF plot of the consensus score (k = 2–9). (**E**) Kaplan–Meier curves demonstrate survival differences based on the GSE72094 cohorts for Cluster-C1 and Cluster-C2. (**F**) PCA shows a remarkable difference in transcriptomes between the two clusters. (**G**–**L**) Comparing the clinicopathological characteristics of patients in Cluster-C1 and Cluster-C2 in the GSE72094 cohort, including age (**G**), gender (**H**), stage (**I**), *EGFR* mutation status (**J**), *KRAS* mutation status (**K**), and *STK11* mutation status (**L**). (**M**) The heat map shows the activation states of biological pathways in two clusters, with red representing the active pathway and blue representing the inhibitory pathway. (**N**) The Violin plot shows the difference in ESTIMATE score, immune score, stromal score, and TumorPurity in two clusters. (**O**) Boxplot displaying the infiltration level of 22 distinct immune cell subtypes in two clusters. (**P**) Immune-related functions in two clusters. (**Q**) The expression levels of immune checkpoint genes in two clusters. *P < 0.05; **P < 0.01; ***P < 0.001; ns, not significant

GO and KEGG enrichment analyses were utilized based on the identification of 1,441 LICMGs in LA scRNA-seq analysis. Moreover, 1,327 GO terms and 125 KEGG pathways were identified. The first six items of biological process (BP), molecular function (MF), and cellular component (CC) are listed in Fig. 3A, such as GTPase regulator activity, nucleoside-triphosphatase regulator activity, and guanyl-nucleotide exchange factor activity enriched in MF; endocytic vesicle, secretory granule membrane, and the cytoplasmic side of the plasma membrane enriched in CC; positive regulation of cell adhesion, regulation of cell–cell adhesion, and mononuclear cell differentiation enriched in BP. The 20 pathways with the smallest qvalue were selected for visualization (Fig. 3B), and results found that LIMG was mainly enriched in Th1 and Th2 cell differentiation, antigen processing and presentation, and human T-cell leukemia virus 1 infection. Not surprisingly, LICMGs have a significant correlation with immunity.

### Identification of molecular subtypes and analysis of clinical characteristics of LICMGs

LICMGs were used to combine transcriptome data from 442 LA samples of the GSE72094 dataset for unsupervised consensus clustering analysis using the “ConsensusClusterPlus” package. When the k value was 2, the GSE72094 samples were stably classified into two clusters with distinct expression patterns (Fig. 3C, D, **Figure **S2**A)**. Based on k = 2, the GSE72094 samples were divided into two clusters, and by the Kaplan–Meier OS curve, we found that the OS time of Cluster-A patients was appreciably longer than that of Cluster-C2 (Fig. 3E). Furthermore, by PCA analysis, we found that the expression profiles of LICMGs were significantly distinct in the two clusters (Fig. 3F). In addition, stage, smoking status, and *TP53* mutations were evenly distributed between the two subgroups. High age, women, and *EGFR* mutations are mainly concentrated in Cluster-C1, whereas *KRAS* and *STK11* mutations are mainly concentrated in Cluster-C2 (Fig. 3G–L, **Figure **S2**B, C)**.

### LA taxonomy based on LICMGs demonstrates distinct biological characteristics and immune microenvironments

The “GSVA” algorithm analysis demonstrated that most of the pathways were mainly enriched in Cluster-C1 (Fig. 3M) and associated with immune system diseases, including asthma, systemic lupus erythematosus, and autoimmune thyroid disease, and Cluster-C1 was also significantly associated with immunoregulating pathways such as T-cell receptor signaling pathway, natural killer cell-mediated cytotoxicity, and JAK-STAT signaling pathway.

The ESTIMATE algorithm analyses are shown in Fig. 3N. The immune score and stromal score of Cluster-C1 were higher than those of Cluster-C2, and the tumor purity of Cluster-C1 was lower than that of Cluster-C2. Thus, we can speculate that the prognosis of Cluster-C1 would be appreciably better than that of Cluster-C2, which is consistent with the abovementioned survival curve results. In addition, CIBERSORT and ssGSEA results revealed that Cluster-C1 has higher proportions of gamma delta T cells, macrophages M1, macrophages M2, resting dendritic cells, and resting mast cells, and Cluster-C2 has higher proportions of plasma cells, T follicular helper cells, T regulatory cells (Tregs), resting NK cells, activated dendritic cells, and neutrophils (Fig. 3O). For ssGSEA, we found that 27 immune cells differed between the two groups, except for type 2 T helper cells (Fig. 3P). In addition, T cell γδ, macrophages, and neutrophils were consistent with CIBERSORT results. Moreover, most immune checkpoints expression had significant differences between the two clusters (Fig. 3Q). Overexpression *of CD40LG* and *PTPRC* in Cluster-C1 was found **(Figure **S2**D, E)**, which indicated a better prognosis, while the overexpression of *LDHA*, *PVR*, *SIGLEC15*, and *YTHDF1* in Cluster-C2 **(Figure **S2**F–I)** revealed a worse prognosis.

### Establishment of LA immune differential score (LIDscore) based on LICMGs and analysis of clinical characteristics in different subgroups

**Fig. 4:**
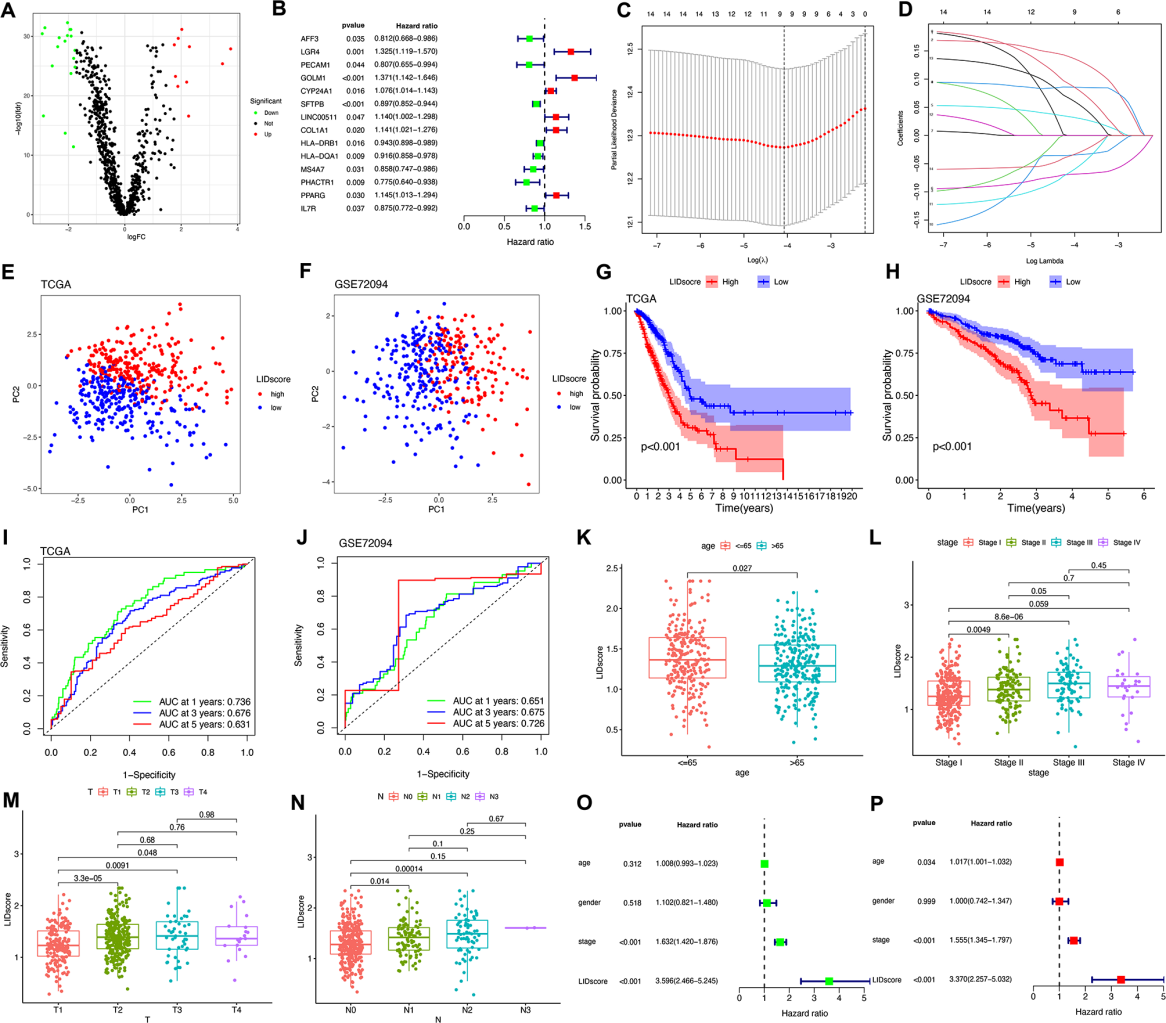
Establishment and verification of the hub-LICMG-based LIDscore model. (**A**) Vocal plot shows risk-related differentially expressed genes. (**B**) Univariate Cox analysis of 14 hub LICMGs. (**C**) LASSO Cox regression model construction, and nine is an optimum parameter. (**D**) LASSO coefficient profiles of 14 risk genes. (**E**, **F**) PCA shows a remarkable difference in TCGA cohort (**E**) and GSE72094 (**F**) cohort transcriptomes between the high- and low-LIDscore subgroups. (**G**, **H**) Kaplan–Meier curves demonstrate survival differences for the two subgroups based on the TCGA (***G***) and GSE72094 cohorts (**H**). (**I**, **J**) The LIDscore model ROC curves in TCGA (**I**) and GSE72094 cohorts (**J**). (**L**–**N**) Comparing the clinicopathological characteristics of patients with high- and low-LIDscore subgroups in the TCGA cohort, including age (**K**), stage (**L**), T stage (**M**), and N stage (**N**). (**O**) Univariate Cox analysis of clinicopathological factors and the LIDscore. (**P**) multivariate Cox analysis of clinicopathological factors and LIDscore

To better classify LICMGs based on co-expression patterns for the TCGA dataset, we performed differential expression analysis on all LICMGs between cancer tissue and normal tissue. In the total 1,441 LICMGs, ten genes were highly expressed in cancer tissues, and 18 genes were expressed highly in normal tissues (Fig. 4A, **Figure **S3**A)**. Further univariate Cox-regression analysis screened out six high-risk and eight low-risk genes (Fig. 4B). Subsequently, further LASSO analysis was performed, and nine hub genes related to prognosis were finally screened, including *LGR4*, *GOLM1*, *CYP24A1*, *SFTPB*, *COL1A1*, *HLA-DQA1*, *MS4A7*, *PPARG*, and *IL7R* (Fig. 4C, D). The LIDscore model is finally constructed by the nine hub genes, with the formula as follows:

$$\begin{array}{l}{\rm{LIDscore}}\,{\rm{ = }}\,{\rm{LGR4*0}}{\rm{.138}}\,{\rm{ + }}\,{\rm{GOLM1}}\,{\rm{*0}}{\rm{.086}}\,{\rm{ + }}\\{\rm{CYP24A1* - 0}}{\rm{.033}}\,{\rm{ + }}\,{\rm{SFTPB}}\,{\rm{*}}\,{\rm{ - 0}}{\rm{.069}}\,{\rm{ + }}\\,{\rm{COL1A1*0}}{\rm{.089 + }}{\rm{HLA}}{\rm{.DQA1* - 0}}{\rm{.035 + }}\,{\rm{MS4A7* - 0}}{\rm{.064 + }}\\,{\rm{PPARG*0}}{\rm{.064 + }}\,{\rm{IL7R* - 0}}{\rm{.0355}}\end{array}$$

Based on this LIDscore model, the LIDscore of each patient was calculated in TCGA, and the median value was used to divide the patients into high- and low-LIDscore subgroups. Through PCA analysis, our model divided the samples of TCGA and GSE72094 (Fig. 4E, F). The Sankey figure demonstrates that the distribution of GSE72094 patients in different clusters, the high- or low-LIDscore groups, and the associated survival status **(Figure **S3**B)**, and through the joint analysis of the different clusters and the high- or low-LIDscore groups, Cluster-C1 group is correlated with the low-LIDscore, and Cluster-C2 group is correlated with the high-LIDscore **(Figure **S3**C)**. The Kaplan–Meier OS curve confirmed that the low-LIDscore group of TCGA patients had significantly longer OS than the high-LIDscore group (Fig. 4G), which was also validated in the GSE72094 dataset (Fig. 4H). In addition, low-LIDscore patients have better outcomes in progression-free survival (PFS) **(Figure **S3**D)**. Time-dependent ROC curve analysis found that the LIDscore was highly effective in predicting a patient’s 1-, 3-, and 5-year overall survival, both in TCGA and GSE72094 (Fig. 4I, J). Clinical data analysis found a significant correlation between the high- and low-score subgroups and age and TNM stage. Surprisingly, the LIDscore of younger patients ( < = 65) was relatively high, but as the stages increased, the LIDscore of patients gradually increased (Fig. 4K–N, **Figure **S3**E, F)**. Univariate and multivariate Cox-regression analyses of survival demonstrated that stage and LIDscore showed an independent prognostic correlation in both analyses (Fig. 4O, P).

### Assessment of the biological and molecular characteristics of LIDscore

**Fig. 5:**
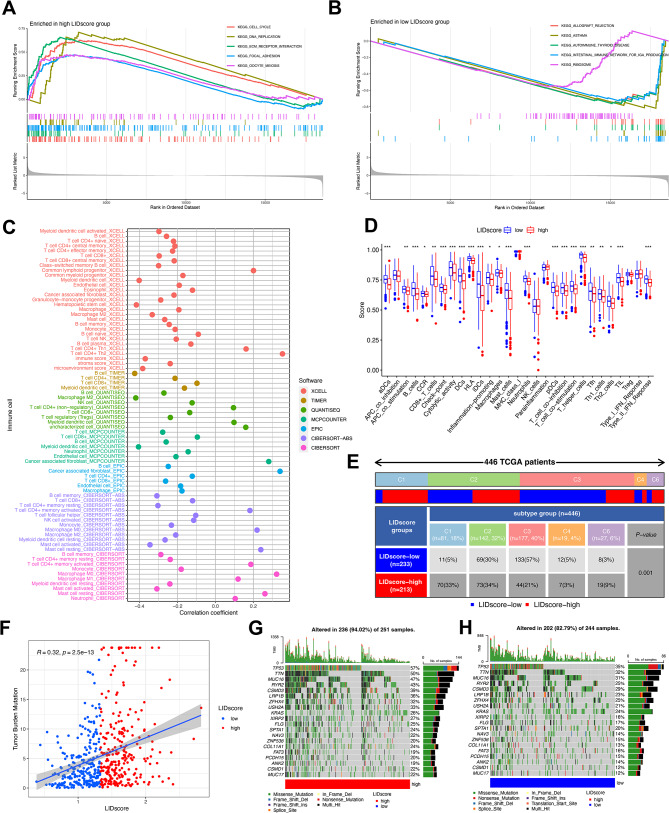
The tumor microenvironment characteristics between LIDscore subgroups. (**A**, **B**) GSEA plots show the gene sets of the TCGA cohort enriched in high- (**A**) and low-LIDscore (**B**) subgroups. (**C**) XCELL, TIMER, QUANTISEQ, MCP-counter, EPIC, CIBERSORT-ABS, and CIBERSORT algorithms comparing tumor-associated infiltrating immune cells in high- and low-LIDscore subgroups. (**D**) Immune-associated functions in high- and low-LIDscore subgroups. (**E**) Patient proportions between LIDscore subgroups among TCGA immune subtypes. (**F**) Scatter plots coordinated by LIDscore and TMB. (**G**, **H**) The oncoPrint plots demonstrate significantly mutated genes in high- (**G**) and low-LIDscore (**H**) subgroups

GSVA analysis found that the high-LIDscore group was significantly enriched in replication and repair pathways in genetic information processing, such as homologous recombination, mismatch repair, and DNA replication, and the low-LIDscore group was significantly enriched in the immune disease pathways in human diseases, such as primary immunodeficiency, asthma, and autoimmune thyroid disease **(Figure **S4**A)**. In addition, through GSEA analysis, the high-LIDscore group was significantly enriched in cell growth and death pathways in cellular processes, such as cell cycle and oocyte meiosis (Fig. 5A), while the low-LIDscore group enrichment pathways were similar to GSVA results (Fig. 5B).

Infiltration differences calculation with seven TME cell deconvolution algorithms found that anti-tumor immune cells are generally infiltrated in low-LIDscore groups such as NK cells, macrophages, CD8 + T cells, mast cells, neutrophils, and B cells. Although the high-LIDscore group also had anti-tumor immune cell infiltration, it was generally at rest, and the high-LIDscore group was highly associated with cancer-associated fibroblast (Fig. 5C). Furthermore, the ssGSEA score quantified the enrichment of 29 immune features per LA patient in TCGA. Immune cell infiltration was similar to the previous seven, B cells, CD8 + T cells, mast cells, and neutrophils were all significantly enriched in the low-LIDscore group (Fig. 5D). Furthermore, the immune score and stromal score of low-LIDscore group were higher than those of high-LIDscore group, and the tumor purity of low-LIDscore group was lower than that of high-LIDscore group **(Figure **S4**B)**.

To further describe the immune landscape of different subgroups, we utilize six immune subtypes identified by previous generations, which included wound healing (c1), IFN-γ dominant (c2), inflammatory (c3), lymphocyte depleted (c4), immunologically quiet (c5), and TGF-β dominant (c6). The results found that low-LIDscore patients had the most enrichment in the c3 group, and low-LIDscore patients might have a better prognosis (Fig. 5E).

Subsequently, gene mutations in tumor cells in the high- and low-LIDscore subgroups demonstrated that TMB was positively correlated with the LIDscore. The higher the LIDscore, the higher the TMB (Fig. 5F). Moreover, higher TMB was associated with a better prognosis for the patient, although the P-value is 0.082 **(Figure **S4**C)**. Combined analysis with the LIDscore showed that the L-TMB + high-LIDscore group has the worst prognosis, and the median survival of H-TMB + low LIDscore is the longest **(Figure **S4**D)**. Furthermore, among the 20 genes with the highest mutation rates, mutation rates in the high-LIDscore group were all higher than those in the low-LIDscore group, where the difference in mutation rates of *TP53*, *TTN*, *MUC16*, and *RYR2* was greater than 15%, and missense mutations were the most common type of mutation, followed by nonsense mutations (Fig. 5G, H).

### Efficacy assessment of immunotherapy and chemotherapy between different subgroups

**Fig. 6:**
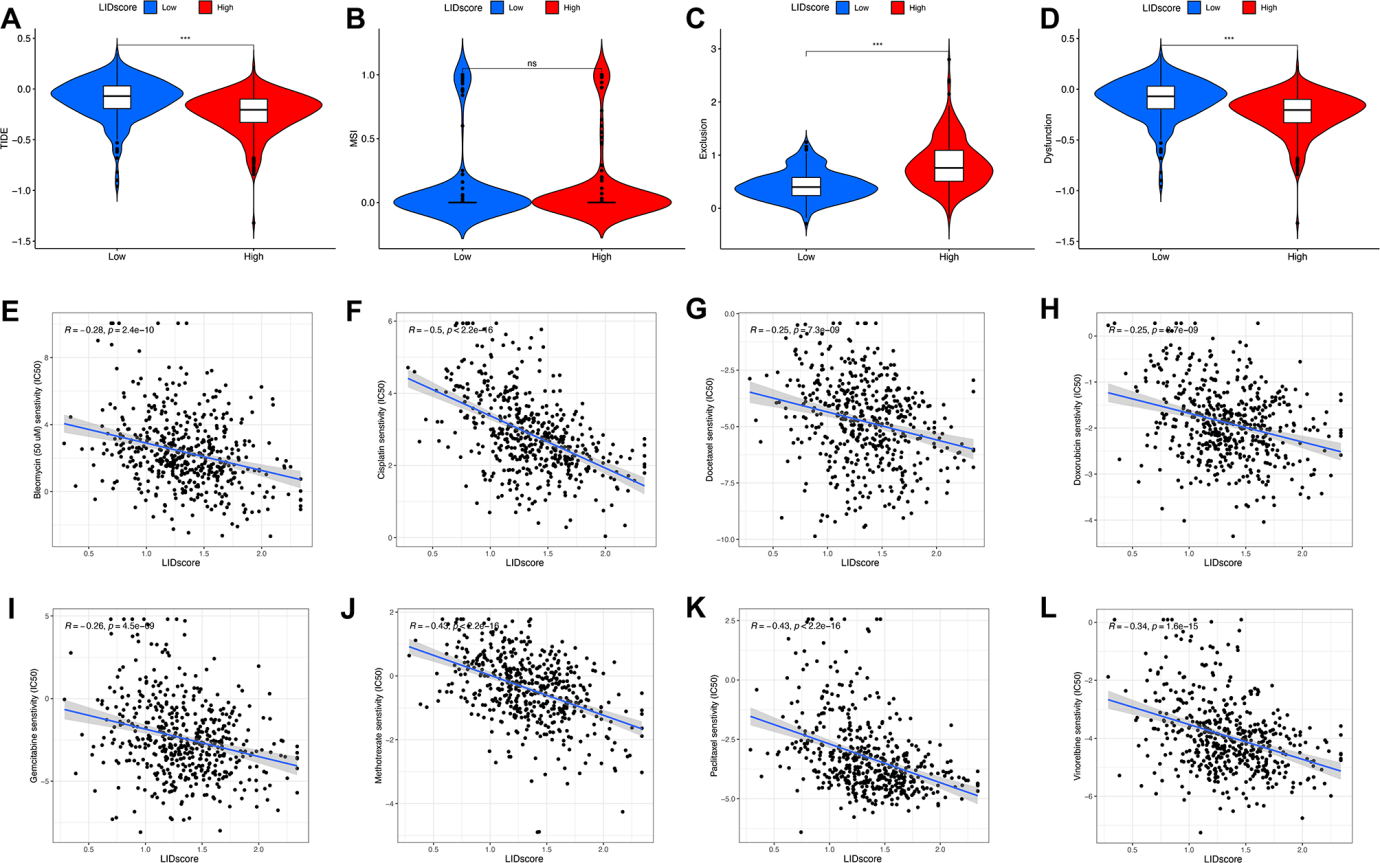
The function of LIDscore in foretelling LA’s immunotherapy response and chemotherapeutic sensitivity. (A–D) TIDE (**A**), MSI (**B**), exclusion (**C**), and dysfunction (**D**) differences between the high- and low-LIDscore subgroups. ∗∗∗P < 0.001, ns, not significant. (**E**–**L**) Correlation between LIDscore and estimated IC50 of bleomycin, cisplatin, docetaxel, doxorubicin, gemcitabine, methotrexate, paclitaxel, and vinorelbine

TIDE score calculation showed a visible distinction in the TIDE score between the two subgroups, the high-LIDscore group had a lower LIDscore (Fig. 6A). Combined with the previous TME score, the LIDscore could predict the benefit of patients from immunotherapy. By comparing the LIDscore with the TIDE score, we found that LIDscore was more sensitive than the TIDE score in predicting the efficacy of immune checkpoint therapy **(Figure **S5). Additionally, dysfunction scores of the low-LIDscore group were significantly higher than those of the high-LIDscore group, while exclusion scores were lower than those of the high-LIDscore group (Fig. 6B–D).

Chemotherapy is still the main treatment for patients with advanced LA. Therefore, we also evaluated the drug sensitivities of several commonly used chemotherapy drugs between high- and low-LIDscore groups, such as bleomycin, cisplatin, docetaxel, doxorubicin, gemcitabine, methotrexate, paclitaxel, and vinorelbine (Fig. 6E–L). We found that the high-LIDscore group generally had higher chemotherapy sensitivity than the low-LIDscore group, which indicates that LIDscore models can help predict not only the effectiveness of immunotherapy but also the sensitivity of chemicals.

### Independent analysis of model genes

**Fig. 7:**
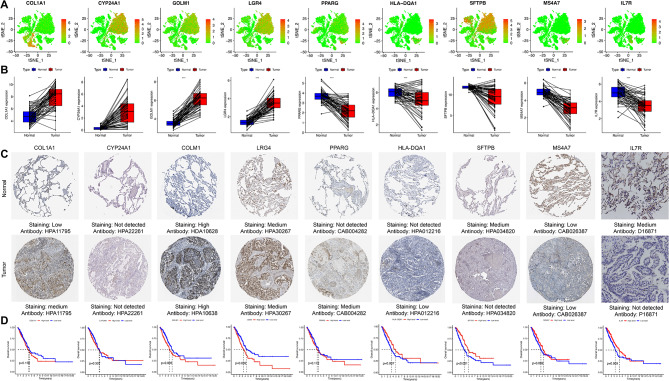
LIDscore model genes analysis. (**A**) Expressions of *LGR4*, *GOLM1*, *CYP24A1*, *SFTPB*, *COL1A1*, *HLA-DQA1*, *MS4A7*, *PPARG*, and *IL7R* in different cell types as classified by the t-SNE algorithm. (**B**) Model genes mRNA expression in 59 paired sample tissues in TCGA. (**C**) Model gene protein expression levels based on immunohistochemical staining. (**D**) Kaplan–Meier curves demonstrate survival differences for high- and low-expression level subgroups based on the model gene

Nine LIDscore model genes were further analyzed. *CYP24A1*, *GOLM1*, *LGR4*, and *SFTPB* are mainly located in epithelial cells, *HLA-DQA1*, *PPARG*, and *MS4A7* are mainly located in macrophages, *COL1A1* is mainly located in smooth muscle cells, and *IL7R* is mainly located in T cells (Fig. 7A). The expression levels of the model genes in nine of the 18 clusters are shown in **Figure **S5. In addition, based on the TCGA dataset, the results of 59 paired samples’ differential mRNA expression of genes are shown in Fig. 7B. *PPARG*, *HLA-DQA1*, *SFTPB*, *MS4A7*, and *IL7R* are lowly expressed in tumor tissues, and *COL1A1*, *CYP24A1*, *GOLM1*, and *LGR4* are highly expressed. Subsequently, their protein expression in normal and tumor tissues was obtained through immunohistochemistry results from the HPA and our immunohistochemical staining. In comparison to normal tissues, tumor tissues have significantly increased levels of COL1A1 and GOLM1 protein expression. The tendency was the opposite for SFTPB and IL7R. However, it was important to highlight that there was no variation in the protein expression levels of CYP24A1, LGR4, PPARG, HLA-DQA1, and MS4A7 (Fig. 7C). Survival analysis demonstrated that high expression of *HLA-DQA1, SFTPB*, and *IL7R* separately had a better prognosis (Fig. 7D). In conclusion, the model’s trend was broadly compatible with the expression of model genes and their proteins.

## Discussion

Current studies have emphasized the significant effect of TIME in the oncogenesis, progression, and metastasis of cancer [12, 34]. In addition, the infiltration level of immune cells in the immune microenvironment determines patient prognosis to varying degrees [35–37]. However, current studies rarely explore the responsiveness of LA to immunotherapy from the differences in the TIME. Single-cell sequencing data allows us to explore differences more accurately in immune cells in the TIME [38]. We identified distinct cellular components through scRNA-seq data analysis and extracted marker genes for all immune cells. Based on this, we combined bulk RNA-seq data, multi-omics, and various clinical data, including survival prognosis, pathological data, somatic mutations, predicted characteristics of two molecular subtypes, and constructed LIDscore models.

We used scRNA-seq data from lung adenocarcinomas to identify cell subsets and extract 1,441 LICMGs. GO and KEGG enrichment analysis displayed that the LICMGs were highly correlated with immune system-related pathways. Subsequently, 1,441 LICMGs identified two molecular subtypes, an immune activation subtype named Cluster-C1 and a metabolically related subtype named Cluster-C2. The medium level of immune infiltration in Cluster-C1 is visibly higher than that in Cluster-C2, with higher anti-tumor immune cells, such as activated CD4+, CD8 + T cells, activated B cells, NK cells, macrophages, gamma delta T cells, and lower cancer-promoting immune cell infiltration of Treg cells. In addition, immune checkpoint genes in the Cluster-C1 group generally have a higher expression level. This result indicated that patients in Cluster-C1 have a better prognosis.

Cluster-C2 is a special subtype of LA characterized by a high enrichment level of pathways related to metabolism, with the most significant processes being amino acid metabolism and carbohydrate metabolism. Current studies suggest that metabolic reprogramming significantly maintains abnormal tumor proliferation, especially when nutrition is limited [39]. High levels of amino acid synthesis can meet the needs of rapid tumor proliferation. Moreover, Cluster-C2 also has a generally low level of immune cell infiltration, in addition to cancer-promoting Treg cells. The high metabolic characteristics of this low immune cell infiltration background may be the main reason for the poor prognosis of Cluster-C2 patients. Research suggests that metabolic reprogramming and immune escape are the main features of malignant tumors [40]. Given Cluster-C2’s special immune and metabolic background, metabolic therapy to adjust its metabolic status to normal levels may be an effective treatment for such patients.

Then we combined LICMGs with the TCGA dataset to screen for differential genes and found the core genes related to immune differences through univariate and lasso cox regression analysis. Nine hub genes, *LGR4*, *GOLM1*, *CYP24A1*, *SFTPB*, *COL1A1*, *HLA-DQA1*, *MS4A7*, *PPARG*, and *IL7R*, were used to construct a LIDscore model, and the LIDscore of each patient in the TCGA was calculated; patients were divided the high- and low-LIDscore subgroups. We found that patients with low-LIDscore had a better prognosis, which was also validated in GSE72094, demonstrating LIDscore as a valid prognostic marker. Furthermore, we discovered by GSVA and GSEA enrichment analysis that the high-LIDscore group was mostly enriched in cellular processes and genetic information processing. By contrast, the low-LIDscore group was significantly enriched in immunological illnesses. Further examination of the immunological microenvironment of the two groups reveals that the low-LIDscore group is highly enriched with various immune cells. According to large-scale studies, higher tumor-infiltrating lymphocyte levels have been linked to fewer recurrences and better overall survival [41, 42]. This may be the primary reason the low-LIDscore group has a better prognosis than the high-LIDscore group. In the examination of immunological subtypes, we discovered that the low-LIDscore group was primarily concentrated in C3, and earlier research has indicated that C3 had the best prognosis.

The total amount of detected somatic mutations per megabase is known as the tumor mutational burden (TMB) [43]. A non-small-cell lung cancer study showed that the patient would respond to immunotherapy better with more mutations in the tumor tissue [44], which is consistent with our study. In addition, we discovered that TMB and risk score are directly correlated. We evaluated the 20 genes with the highest ratio of mutant genes in the two groups. We discovered that the proportion of mutated genes in the high-risk group was higher than that in the low-risk group, particularly *TP53*, *TTN*, *MUC16*, and *RYR2*. The levels of immunological checkpoints, interferon-γ signature, and activated T-effectors were all dramatically elevated by *TP53* mutations [45]. High immunogenicity is essential for predicting the outcome of patients receiving immunotherapy, and LA patients with TTN mutation had high immunogenicity [46]. *MUC16* and *RYR2* mutations are associated with reported immunotherapy responses in solid tumors [47, 48]. These findings imply that patients in the high-LIDscore category might respond to immunotherapy more positively than individuals in the low-LIDscore group.

TIDE is a biomarker to evaluate patient response to immune checkpoint medication [31]. We put our conjecture to the test. Unsurprisingly, TIDE scores of high-LIDscore group were lower, meaning patients were more responsive to immunotherapy. After analyzing the causes, we prove that although the low-LIDscore group has more immune infiltration, the proportion of pro-tumor immune cells is still higher. The low-LIDscore group has a larger amount of immune infiltration than the high-LIDscore group, and Treg is also high, suppressing the anti-tumor action of immune cells in the TIME. Further evidence from the T-cell dysfunction scores and the T-cell rejection scores confirms that the high-LIDscore group is more likely to benefit from immunotherapy. Although a higher T-cell exclusion score indicates a greater likelihood of immune evasion, a lower T-cell dysfunction score may enhance ICI responses [31]. At the same time, this study also explored the predictive ability of the LIDscore model for sensitivity to conventional chemotherapy. Patients in the high-LIDscore group were typically more sensitive to common chemotherapy drugs than patients in another group, including bleomycin, cisplatin, docetaxel, doxorubicin, gemcitabine, methotrexate, paclitaxel, and vinorelbine. Therefore, it can be used to guide the use of chemotherapy.

Among the nine of LIDscore model genes, *COL1A1*, *CYP24A1*, *GOLM1*, *LGR4*, and *PPARG* were predictors of unsatisfactory survival, while *HLA-DQA1*, *IL7R*, *MS4A7*, and *SFTPB* were related to reduced prognosis risk. Collagen type I α1 (*COL1A1*), the major component of type I collagen, enhanced oncogenicity on hepatocellular carcinoma (HCC) cells. Down-regulation of *COL1A1* expression can prevent the proliferation, invasion, and formation of tumor spheroids of HCC cells [49]. In addition, increased expression of *COL1A1* promoted the malignant transformation induced by coke oven emission [50]. High expression of the cytochrome P450 family 24 subfamily A member 1 (*CYP24A1*) is linked to poor prognosis of resection of LA, and it has carcinogenic properties mediated by raising RAS signaling [51]. Golgi membrane protein 1 (*GOLM1*) affects the biology of non-small cell lung cancer (NSCLC) and enhances the aggressiveness of NSCLC by inhibiting the formation of P53 tetramer [52]. Leucine-rich repeat-containing G protein-coupled receptor 4 (*LGR4*) is significantly expressed in numerous cancer types and is linked to a worse patient prognosis [53, 54]. Receptor PPARG inhibits the growth of cancer cells, including NSCLC [55]. However, it has a pro-tumor effect on cells in the microenvironment, especially myeloid cells [56]. The aggressiveness of Keap1-deficient LA may be caused by abnormal RSPO3-LGR4 signaling [57]. HLA-DQA1 is a para chain of the HLA class II alpha chain associated with considerably longer OS [58], and an immune gene associated with antigen presentation, with a decrease indicating an immunosuppressive microenvironment and invasive disease [59, 60]. Members of the MS4A family are important players in various clinical situations, including neurodegeneration, infectious illnesses, and cancer [61]. Human tissue macrophages and monocyte-derived macrophages both expressed the MS4A4A/MS4A6A/MS4A7/MS4A8 cluster [62]. This cluster may interact with PRRs to coordinate type 1 immunity by assisting macrophages in their “sensing” role [63]. SFTP inhibits lung cancer progression by suppressing secretory arachidonic acid production [64]. Previous results are generally consistent with our results, but additional research is required to confirm the biological roles of the hub genes above.

The model we constructed has certain advantages. We screened out immune cells in the tumor microenvironment by single-cell sequencing, and further screened out differentially expressed genes in immune cells for subsequent model construction. Compared with traditional prognostic models built only on immune-related genes obtained from whole-transcriptome sequencing, our model has the advantages of higher reliability and greater pertinence [65, 66]. And the deep combination of this model with immunity has significant advantages in predicting the efficacy of immunotherapy. But Our study had some limitations. First, there is heterogeneity in the two largest LA databases and our scRNA-seq data, although many algorithms were utilized to minimize potential batch effects. Second, further multi-center and prospective clinical research investigations are still needed to support our theory. Third, additional experimental validation in vivo and in vitro is needed, including simply bioinformatics analysis and validation at the protein and gene expression levels. Furthermore, since immunohistochemistry results cannot distinguish cell types, the expression levels of the different model genes in cell subtypes still need further study.

## Conclusions

In this study, we systematically evaluated the tumor heterogeneity of LA, performed molecular typing of LA based on 1,441 LICMGs, obtained two subtypes related to immune activation and metabolism, and constructed a LIDscore model with nine LICMGs, which could predict the prognosis of LA patients, evaluate the clinical relevance and biological and molecular characteristics differences, and help select LA patients who respond more strongly to immunotherapy and chemotherapy.

### Electronic supplementary material

Below is the link to the electronic supplementary material.

**Additional file 1. Figure S1**. (A) The dimensionality of the 15 PCs were reduced using the tSNE algorithm and successfully yielded four cell clusters whose top 20 markers genes are shown in dot plots. (B) Heatmap demonstrating the differentially expressed genes across the 10 clusters. The expressions of top 20 marker genes from each cluster are shown. The colors from purple to yellow indicate the gene expression levels from low to high. **Figure S2**. (A) Relative change in area under CDF curve. (B?C) Comparing the clinicopathological characteristics of patients in Cluster-A and Cluster-B in the GSE72094 cohort, including smoking status (B) and tp53 mutation status(C). (D?I) Kaplan-Meier plots of overall survival difference between tumors with high and low level of different immune cells infiltration or immune relating genes expression. **Figure S3**. (A) heatmap shows risk-related differentially expressed genes. (B) Sankey figure shows the changes of cluster, LIDscore level and survival outcomes. (C) Differences in LIDscore among two clusters in GSE72094 cohorts. The Kruskal-Wallis test was used to compare the statistical difference. (D) Kaplan?Meier curves demonstrate progression free survival differences for the two subgroups based on the TCGA cohorts. (E, F) Comparing the clinicopathological characteristics of patients with high- and low-LIDscore subgroups in the TCGA cohort, including gender (E) and M stage (F). **Figure S4**. (A) GSVA enrichment analysis shows the activation differences of biological pathways in low- and high-LIDscore groups. The heatmap visualized these biological pathways, and red represented activated pathways and blue represented inhibited pathways. (B) The Violin plot shows the difference in ESTIMATE score, immunescore, stromal score, and TumorPurity in low- and high-LIDscore groups. (C) Comparison of overall survival in the high- and low-LIDscore groups. (D) The Kaplan-Meier survival curve reflecting the interrelationship among TMB, LIDscore and patient survival. **Figure S5**. Performance contrast between of LIDscore, TIDE and TIS in forecasting 3-year OS on TCGA cohort. **Figure S6**. Bubble plot showing the expression of LICMGs included in the risk score model in various cell types. Bubble intensity of colour indicates the average expression in a particular cluster and bubble size represents the percent of cells expressing the gene in that cluster.

**Additional file 2. Supplementary Table 1**. Differentially expressed genes of all type of cells

**Additional file 3. Supplementary Table 2**. Differentially expressed genes of all immune cells

**Additional file 4. Supplementary Table 3**: Results of GO pathyway analysis of LICMGs

**Additional file 5. Supplementary Table 4**: Results of KEGG pathyway analysis of LICMGs

**Additional file 6. Supplementary Table 5**.Results of GSVA analysis in cluster-A and clusier-B

**Additional file 7. Supplementary Table 6**: Results of univariate cox regression analysis on survival related LICMGs

**Additional file 8. Supplementary Table 7**:?Results of LASSO Cox regression analysis.

**Additional file 9. Supplementary Table 8**. Univariate cox analysis on clinical features

**Additional file 10. Supplementary Table 9**. Results of multivariate cox regression analysis

**Additional file 11. Supplementary Table 10**. Results of GSVA analysis

**Additional file 12. Supplementary Table 11**. Results of GSEA analysis

**Additional file 13. Supplementary Table 12**: Tide results for TCGA-LUAD data

**Additional file 14. Supplementary Table 13**: TISgene

**Additional file 15. Supplementary Material 15**

## Data Availability

All data generated or analyzed during this study are included in this published article. We downloaded the corresponding public data resources from the TCGA repository, (https://portal.gdc.cancer.gov/) (TCGA cohort, n = 594) and the Gene Expression Omnibus (http://www.ncbi.nlm.nih.gov/geo/) (LUAD cohort, GSE72094, n = 442).
