# Supplementary material for: Molecular subtyping based on immune cell marker genes predicts prognosis and therapeutic response in patients with lung adenocarcinoma
Source: BMC Cancer. 2023 Nov 24;23:1141. doi: 10.1186/s12885-023-11579-7 (PMC10668343; doi:10.1186/s12885-023-11579-7)
Supplement: Supplementary file 1 — Additional file 1. Figure S1. (A) The dimensionality of the 15 PCs were reduced using the tSNE algorithm and successfully yielded four cell clusters whose top 20 markers genes are shown in dot plots. (B) Heatmap demonstrating the differentially expressed genes across the 10 clusters. The expressions of top 20 marker genes from each cluster are shown. The colors from purple to yellow indicate the gene expression levels from low to high. Figure S2. (A) Relative change in area under CDF curve. (B?C) Comparing the clinicopathological characteristics of patients in Cluster-A and Cluster-B in the GSE72094 cohort, including smoking status (B) and tp53 mutation status(C). (D?I) Kaplan-Meier plots of overall survival difference between tumors with high and low level of different immune cells infiltration or immune relating genes expression. Figure S3. (A) heatmap shows risk-related differentially expressed genes. (B) Sankey figure shows the changes of cluster, LIDscore level and survival outcomes. (C) Differences in LIDscore among two clusters in GSE72094 cohorts. The Kruskal-Wallis test was used to compare the statistical difference. (D) Kaplan?Meier curves demonstrate progression free survival differences for the two subgroups based on the TCGA cohorts. (E, F) Comparing the clinicopathological characteristics of patients with high- and low-LIDscore subgroups in the TCGA cohort, including gender (E) and M stage (F). Figure S4. (A) GSVA enrichment analysis shows the activation differences of biological pathways in low- and high-LIDscore groups. The heatmap visualized these biological pathways, and red represented activated pathways and blue represented inhibited pathways. (B) The Violin plot shows the difference in ESTIMATE score, immunescore, stromal score, and TumorPurity in low- and high-LIDscore groups. (C) Comparison of overall survival in the high- and low-LIDscore groups. (D) The Kaplan-Meier survival curve reflecting the interrelationship among TMB, LIDscore and pati [file 12885_2023_11579_MOESM1_ESM.docx]

**Figure S1.** (A) The dimensionality of the 15 PCs were reduced using the tSNE algorithm and successfully yielded four cell clusters whose top 20 markers genes are shown in dot plots. (B) Heatmap demonstrating the differentially expressed genes across the 10 clusters. The expressions of top 20 marker genes from each cluster are shown. The colors from purple to yellow indicate the gene expression levels from low to high.


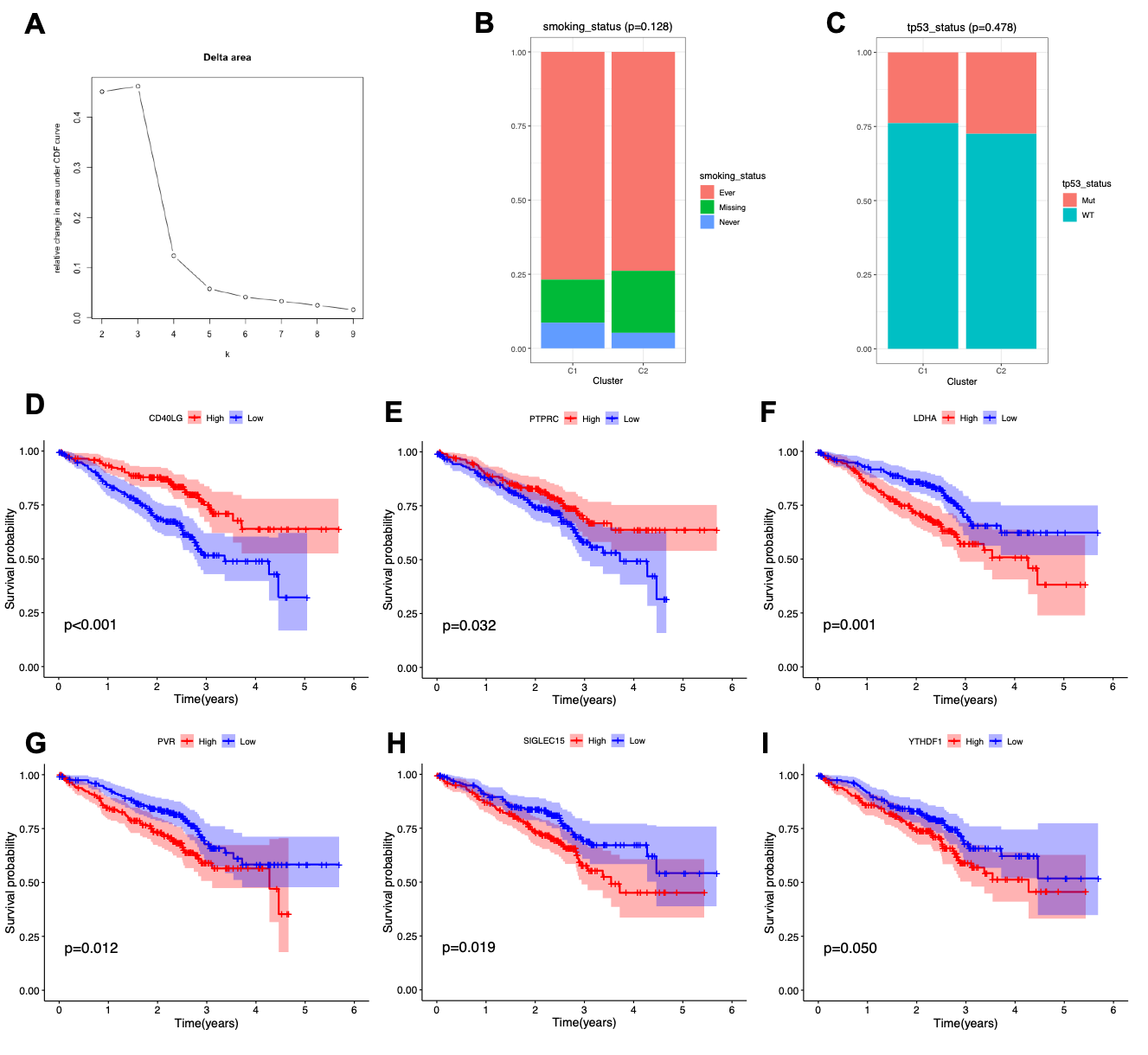


**Figure S2.** (A) Relative change in area under CDF curve. (B–C) Comparing the clinicopathological characteristics of patients in Cluster-A and Cluster-B in the GSE72094 cohort, including smoking status (B) and tp53 mutation status(C). (D–I) Kaplan-Meier plots of overall survival difference between tumors with high and low level of different immune cells infiltration or immune relating genes expression.


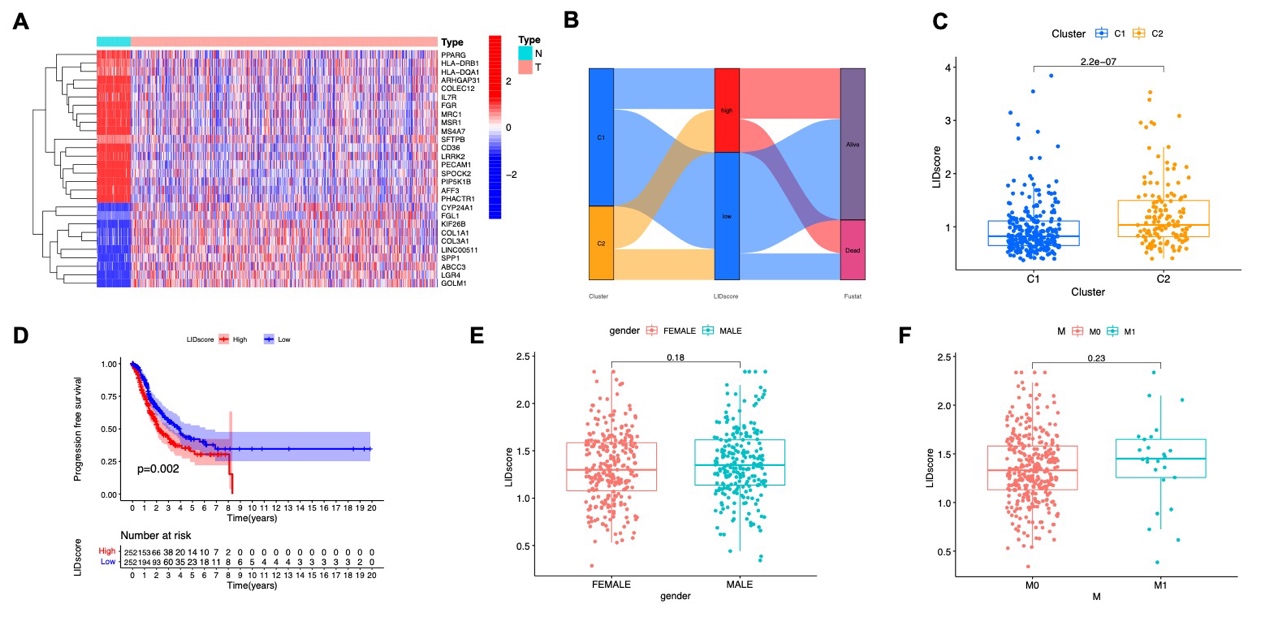


**Figure S3.** (A) heatmap shows risk-related differentially expressed genes. (B) Sankey figure shows the changes of cluster, LIDscore level and survival outcomes. (C) Differences in LIDscore among two clusters in GSE72094 cohorts. The Kruskal-Wallis test was used to compare the statistical difference. (D) Kaplan–Meier curves demonstrate progression free survival differences for the two subgroups based on the TCGA cohorts. (E, F) Comparing the clinicopathological characteristics of patients with high- and low-LIDscore subgroups in the TCGA cohort, including gender (E) and M stage (F).

**Figure S4.** (A) GSVA enrichment analysis shows the activation differences of biological pathways in low- and high-LIDscore groups. The heatmap visualized these biological pathways, and red represented activated pathways and blue represented inhibited pathways. (B) The Violin plot shows the difference in ESTIMATE score, immunescore, stromal score, and TumorPurity in low- and high-LIDscore groups. (C) Comparison of overall survival in the high- and low-LIDscore groups. (D) The Kaplan-Meier survival curve reflecting the interrelationship among TMB, LIDscore and patient survival.

**Figure S5.** Performance contrast between of LIDscore, TIDE and TIS in forecasting 3-year OS on TCGA cohort.

**Figure S6.** Bubble plot showing the expression of LICMGs included in the risk score model in various cell types. Bubble intensity of colour indicates the average expression in a particular cluster and bubble size represents the percent of cells expressing the gene in that cluster.
